# A Concise History of the Principal Discoveries in Anatomy

**Published:** 1799-10

**Authors:** 


### *A concise History of the principal Discoveries in Anatomy.*

[Continued from our last Number, pp. 160—168.]

§ 18. THE gradual progress towards the most interesting of all anatomical discoveries may be traced from the method of explaining the *smaller circulation of the blood through the lungs*, which was adopted by many anatomists towards the end of the sixteenth century. Much depended here on a due examination of the septum of the ventricles of the heart, which GALEN considered as perforated, or at least supposed that there were pits in it large enough to form the third ventricle of the heart. Even BERENGAR perceived the incongruity of this idea, while he found the septum so solid, and the perforations in the human heart so imperceptible, that he expressly declares it to be next to impossible that the blood could exude, through this septum, from the right to the left ventricle of the heart. As this notion prevailed, anatomists were consequently induced to conjecture, that the vena cava likewise originated from the heart; and hence VESALIUS so obstinately maintained the imperforation of the septum. If therefore the vena cava arose in the liver, and conducted the blood to the heart, in this case the aorta, which besides the vital spirits contains blood also, must either receive this blood from the pulmonary artery, after it had passed through the lungs (this circulation, however, was then unknown), or there remained no other method of accounting for it, than by admitting the exudation of the blood through that septum. The ingenious LAGUNA paid attention to this circumstance, when he maintained that the septum is perforated, and that part of the blood circulated from the right into the left ventricle of the heart, while another part flowed through the pulmonary artery into the lungs, and served for their nourishment. Hence MONAVIUS wrote to CRATO, that PIGAFETTA, a pupil of FALLOPIUS, had publicly maintained in the University of Heidelberg, that the septum of the ventricles of the heart is impermeable; an opinion which would be branded with heresy by the German physicians of those times! Perhaps these authors were misled, by comparing the structure of the heart in the lower animals with that of man, to suppose that the aperture of the foramen ovale in man tinued even after birth, and consequently to admit the perforation of the septum.

Next to Vesalius, MICHAEL SERVETO was the first who delivered the opinion relative to the complete impermeability of the septum, and ingeniously applied it to explain the circulation of the blood through the lungs; a discovery, the first traces of which appear in the writings of this author. He says, for instance, "the vital spirit of the arteries penetrates, through their anastomoses with the veins, into the latter; for, according to the previous assertion of Vesalius, every vein, in the different parts of the human body is most intimately connected with an artery. It is impossible that the blood can pass through the septum from the right into the left ventricle of the heart, because the former is quite impermeable: hence it must pass through the lungs; here it receives fresh vital spirits from the atmospheric air, and thus it again returns from the lungs to the heart." That the purpose of the pulmonary artery cannot be that alone of nourishing the lungs, Serveto concludes from this circumstance, in particular, that the artery in question is uncommonly large and wide in proportion to its vein, that it is accompanied throughout by the vein, and that there are other vessels designed by Nature for the support of the lungs. Nor is it conceivable, that the accession of vital spirit takes place in either of the two ventricles of the heart, as neither of them is sufficiently capacious for that purpose." *—In this passage, therefore, we recognize the first germ for discovering the circulation of the blood through the lungs. It was written about the year 1552, and the work of Serveto appeared in 1553.—It has indeed been maintained from a cotemporary work, written by JAMES RUEFF, a surgeon, at Zurich, and published in 1554, that he discovered the great circulation of the blood; but it can be attributed only to their total ignorance of literary history, and their defective explanations of difficult passages, that some French surgeons preposterously endeavoured to wrest the laurel from the immortal HARVEY, † and bestow it upon their countryman, Rueff. The passage to which GARENGEOT alludes, treats merely of the distribution of vital spirits through the whole body, by the arteries: ‡ It is, however, unnecessary to enter into farther particulars respecting this subject; as PORTAL § has amply refuted the last-mentioned writer.

[To be resumed in our next Number.]

* *Servet.* "*Restitut. Christianism.*" Lib. v. p. 169. Edit. 1790

† *Garengeot* "*Spanchnologie:*" Vol. II. p. 156. & seq.

‡ *Rueff* "*de Conceptu et Generat.*" Lib. i. cap. iv. f. 6. b.

§ *Portal* "*Hist. de l'Anatom.*" Vol. I. p. 515.

